# Guillain-Barre Syndrome Following a Snakebite: A Case Report and Review of Literature

**DOI:** 10.7759/cureus.5278

**Published:** 2019-07-30

**Authors:** Sajid Hameed, Mubashar Memon, Sara Khan

**Affiliations:** 1 Neurology, Aga Khan University, Karachi, PAK

**Keywords:** snake bite, guillain-barre syndrome

## Abstract

Guillain-Barre syndrome (GBS) is preceded by a respiratory or gastrointestinal illness in more than 50% of the patients. A rare association of GBS with a preceding snakebite is reported in the literature in only four previous cases. We present a case report of a patient diagnosed with GBS following the bite of a yellow-bellied sea snake *(Hydrophis platurus)* and a brief review of the available literature.

## Introduction

Guillain-Barre syndrome (GBS) is an acute-onset acquired polyneuropathy with an annual incidence of 0.5-2 cases per 100,000 [1]. More than half of the patients have a history of preceding respiratory or gastrointestinal illness. An association of GBS with preceding snakebite is rare and has been reported in the literature in only four previous cases. We present a case of GBS following snakebite and a brief review of the available literature.

## Case presentation

A 21-year-old male, fisherman by profession, presented with a three-week history of progressive generalized weakness, diplopia, decreased appetite and lethargy with difficulty in performing activities of his daily living. For the past three days, he was having a high-grade fever and shortness of breath. He was managed in the medicine department with the diagnosis of aspiration pneumonia with type-1 respiratory failure. The patient reported that six weeks ago, he was bitten by a yellow-bellied sea snake *(Hydrophis platurus)* on his left palm during fishing. No anti-venom was administered at that time. Neurology team was consulted on the second day of admission.

On physical examination, he had a blood pressure of 90/60 mmHg, a pulse of 85 per minute and a temperature of 98.2 F. He was on continuous non-invasive ventilation (NIV) support for his hypoxia. He had a well-demarcated clean healing wound of around 2-cm in diameter on his left palm. He was awake and oriented with intact higher mental functions. Pupils were of 3-mm bilaterally and reactive to light and accommodation. Right lateral rectus palsy was noted. There was no facial muscle weakness and tongue was central on protrusion. His speech had a hyper-nasal quality. Movements of soft palate and uvula were absent on testing with an absent gag reflex. On motor examination, he had normal bulk without fasciculation. Muscle tone was mildly reduced in all limbs. In both upper limbs, he had muscle strength of Medical Research Council (MRC) grade 3/5 in proximal muscle groups and MRC grade 4/5 in distal muscle groups, while in lower limbs he had MRC grade of 4/5 in both proximal and distal muscle groups. Deep tendon reflexes were absent in all four limbs. Sensory examination was unremarkable.

Investigations

Initial laboratory investigations (on day one) are shown in Table 1. 

**Table 1:** Laboratory investigations

Test	Result	Reference
Hemoglobin	15.6 g/dL	12.3 - 16.6 g/dL
White blood cell count	18.2 x 10E9/L	4.8-11.3 x 10E9/L
Neutrophils	85.8 %	34.9 - 76.2 %
Lymphocytes	4.4 %	17.5 - 45 %
Platelet count	544 x 10E9/L	154 - 433 x 10E9/L
Procalcitonin	1.67 ng/mL	Positive > 2.0 ng/mL
Magnesium	1.8 mg/dL	1.6 - 2.6 mg/dL
Blood urea nitrogen	32 mg/dL	6 - 20 mg/dL
Creatinine	1.3 mg/dL	0.9 - 1.3 mg/dL
Sodium	140 mmol/L	136 - 145 mmol/L
Potassium	3.8 mmol/L	3.5 - 5.1 mmol/L
Chloride	97 mmol/L	98 – 107 mmol/L
Bicarbonate	33.1 mmol/L	20 – 31 mmol/L
Calcium	9.6 mg/dL	8.6 - 10.2 mg/dL
Erythrocyte sedimentation rate	95 mm/1st hour	0-15 mm/1st hour
C-reactive protein	15.59 mg/dL	0 – 0.5 mg/dL

**Figure 1:**
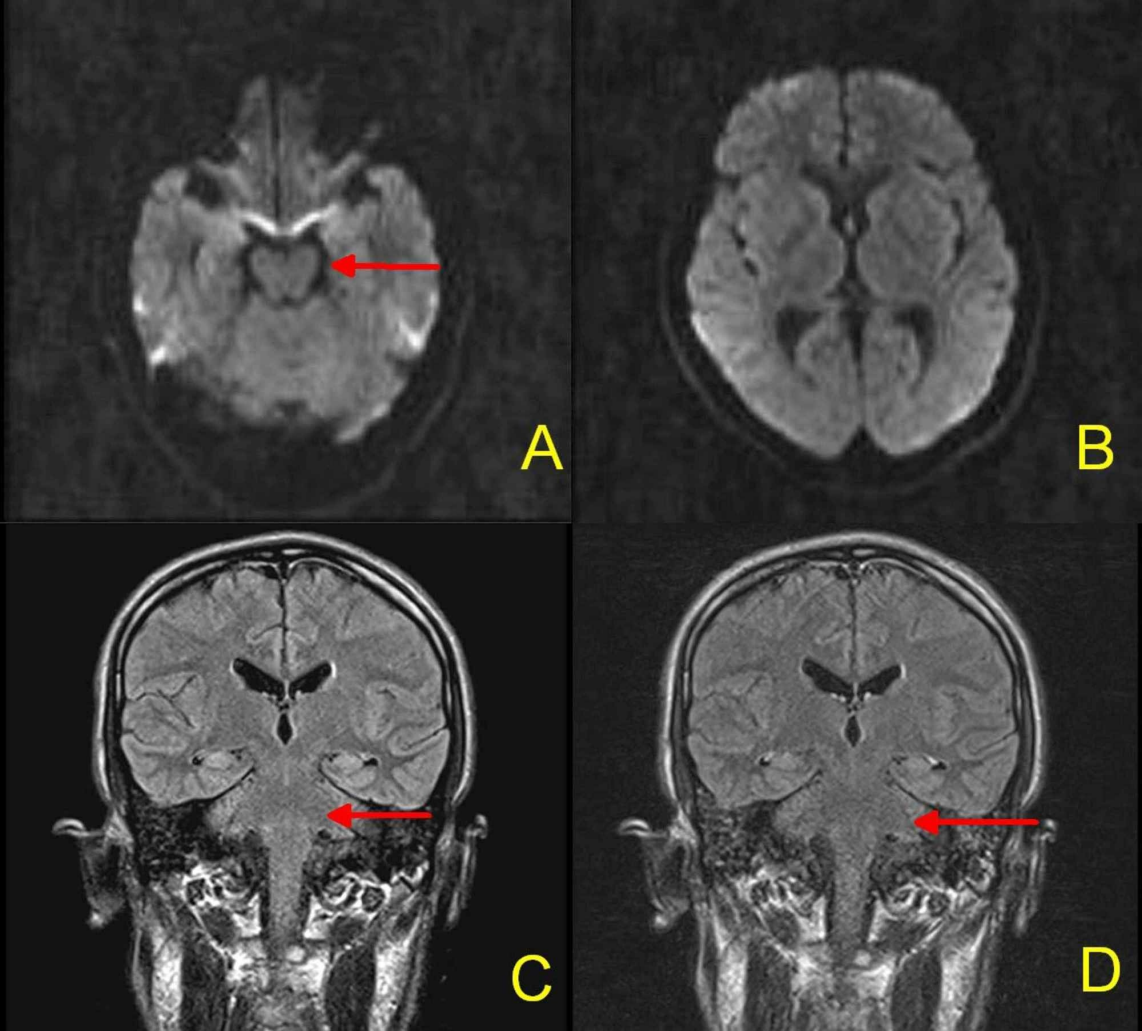
MRI Brain: No acute ischemic stroke on DWI sequences (Figure 1A, 1B). No abnormal signals on pre-contrast ( Figure 1C) or post-contrast FLAIR sequence (Figure 1D). Red arrows indicate brainstem, which is normal MRI = Magnetic resonance imaging; DWI = Diffusion-weighted imaging; FLAIR = Fluid-attenuated inversion recovery

Our differential diagnosis indicated either a brainstem stroke or demyelination. Cranial magnetic resonance imaging (MRI) was negative for an acute brainstem stroke or abnormal contrast enhancement (Figure 1).

On the third day of admission, electromyography and nerve conduction studies (EMG/NCS) were performed. These revealed normal sensory nerve conduction, while motor amplitudes of the bilateral peroneal, right median, and ulnar nerves were reduced without a conduction block. In the clinical context, the patient was diagnosed with an acute motor axonal neuropathy (AMAN) variant of GBS.

Management

The patient was initially managed with continuous NIV and broad-spectrum antibiotics for aspiration pneumonia.

On the third day of admission, plasmapheresis was initiated after obtaining the EMG/NCS results. The decision of plasmapheresis over IVIG was made based on the patient's preference. Vital capacity was monitored regularly. After the third session of plasmapheresis (on day five), he developed severe respiratory distress and SaO2 dropped to 65% despite NIV following which he was intubated. The remaining two sessions of plasmapheresis were done in the intensive-care unit (ICU). On the seventh day of admission, the final (fifth) session of plasmapheresis was done. On day nine, the patient was extubated.

From days 9-14, the patient underwent regular inpatient physiotherapy and rehabilitation. On day 14, he was discharged home in a stable condition with a nasogastric tube (NGT) in place.

Follow-up

The patient was stable on the first follow up at the clinic (after three weeks of being discharged from the hospital). The NGT was removed and he was taking food orally. Muscle strength was of MRC grade 3/5 in all four limbs. Right lateral rectus palsy was present.

On the second clinic follow up (after four months of being discharged from the hospital), the patient regained his muscle strength to MRC grade 5/5 in all four limbs and was ambulating without difficulty. The right lateral palsy persisted.

## Discussion

Guillain-Barre syndrome is an acute autoimmune-mediated polyneuropathy that commonly presents with bilateral symmetrical ascending flaccid paralysis. However, variations in the clinical presentation of GBS do exist, as in our case. About 70% of patients with GBS have a preceding event, most commonly a respiratory or gastrointestinal illness. Additionally, GBS is reported to be associated with surgery, cancer, and vaccines [1]. Only a few case reports suggest an association of a snakebite with GBS [2-4].

In 1996, Chuang et al. reported the first case of GBS in association with a snakebite [2]. The patient presented with progressive quadriparesis, autonomic dysfunction, and cerebrospinal fluid (CSF) cytoalbuminologic dissociation, four weeks after the bite of a Formosan krait *(Bungarus multicinctus)*. EMG/NCS revealed sensorimotor axonal-type polyneuropathy. The patient improved with subsequent treatment (plasmapheresis and methylprednisolone of 500 mg/day for five days) and rehabilitation.

The second case was reported by Srivastava et al. in 2010 [3]. In this case, the patient presented with bilateral paresthesia in the upper limbs and quadriparesis five weeks after the snakebite. The snake species could not be identified. EMG/NCS revealed motor and sensory neuropathy-primarily suggestive of demyelination with secondary axonal degeneration. The patient improved with plasmapheresis.

In 2011, Neil et al. [4] reported the third case of GBS following the bite of the snake,* Vipera aspis*. The patient presented with paraesthesia and quadriparesis after 12 days of snakebite. The authors went one step further and demonstrated cross-reactivity between venom proteins and neuronal GM2 gangliosides (molecular mimicry), postulating a potential immunological basis for this association rather than direct venom toxicity.

The fourth case was reported in 2014 by Neto et al. from Brazil [5]. In this case, the patient presented with quadriparesis and areflexia following the bite of Rattlesnake *(Crotalus sp.)* two weeks back. The patient improved with intravenous immunoglobulin (IVIG) and rehabilitation. EMG/NCS performed on follow-up (on the 60th day) was consistent with sensory and motor axonal polyradiculoneuropathy.

To the best of our knowledge, this is the fifth case of GBS associated with a snakebite. All the patients presented later, from 12 days [5] to five weeks [3], following the snake bite indicating an immune-related etiology rather than direct effects of the toxin, as suggested by Neil et al. [4]. Another possibility was that the anti-venom may be associated with GBS rather than the snake venom itself. However, two of the patients, including ours, didn’t receive the anti-venom [4]. All the patients improved with treatment and were able to perform activities of daily living on follow-up visits. One patient received IVIG and three patients received plasmapheresis, out of which one also received additional methylprednisolone. The treatment of the remaining patient is unknown. Both IVIG and plasmapheresis are equally effective in the treatment of GBS, while steroids have no proven role [1].

Our case was slightly different in that the snake was native to water and the polyneuropathy was of pure motor type (AMAN variant of GBS), while all other cases presented with mixed sensorimotor polyneuropathies. All the previous case reports have been summarized in Table 2.

**Table 2:** Case reports of Guillain-Barre syndrome following snake bite. EMG/NCS = Electromyography and nerve conduction studies; IVIG = intravenous immune globulin

Authors (Country & year)	Snake species	Clinical features	Time duration from snakebite to Presentation	Antivenom received	EMG/NCS	Treatment
Chuang et al. [2] (Taiwan, 1996)	Formosan krait (Bungarus multicinctus)	Quadriparesis, facial weakness, and autonomic dysfunction	Four weeks	Yes	Sensorimotor axonal-type polyneuropathy.	Plasmapheresis; Methylprednisolone, 500 mg/d for five days
Srivastava et al. [3] (India, 2010)	---	Quadriparesis and paresthesia in upper limbs.	Five weeks	Yes	Sensorimotor polyneuropathy—primarily suggestive of demyelination with secondary axonal degeneration.	Plasmapheresis
Neil et al. [4] (France, 2011)	Viper (Vipera Aspis)	Paresthesia in all four extremities, gait ataxia, and quadriparesis	12 days	No	Sensorimotor polyneuropathy - primarily suggestive of demyelination with conduction block	----
Neto et al. [5] (Brazil, 2014)	Rattlesnake (Crotalus sp.)	Quadriparesis	Two weeks	Yes	Sensorimotor axonal polyradiculoneuropathy.	IVIG

Snakebite is a common condition, but a neglected one [6]. Although exact data is lacking, the annual estimates vary from 4.5 to 5.4 million snakebites, 1.8 to 2.7 million envenomings, and 81,000 to 138,000 deaths according to the World Health Organization [7]. Despite these huge numbers, only a handful of cases of GBS following snakebite are reported in the literature. We can assume that many cases would have been missed due to under-reporting. Nonetheless, further studies are needed to confirm the association between GBS following a snakebite.

## Conclusions

The temporal association of the snakebite with GBS in all the aforementioned cases suggest a possible association. Patients with a snakebite should be observed for new-onset flaccid weakness for multiple weeks and if present, it should be addressed urgently. The prognosis is excellent if timely interventions are performed.
